# Influence of Crestal Mucosal Thickness on the Accuracy of Gingival Former Selection in Single-Tooth Implant Prosthodontics: An Observational Study

**DOI:** 10.7759/cureus.83477

**Published:** 2025-05-04

**Authors:** Anju Jha, Rohit Patil, Samra Ashraf, Naila Perween, Shahinwaz Mulani, Girija Dodamani, Seema Gupta

**Affiliations:** 1 Department of Pediatric and Preventive Dentistry, Patna Dental College and Hospital, Patna, IND; 2 Department of Prosthodontics, Jawahar Medical Foundation's Annasaheb Chudaman Patil Memorial Dental College, Dhule, IND; 3 Department of Prosthodontics, Teerthanker Mahaveer Dental College and Research Centre, Moradabad, IND; 4 Department of Prosthodontics, ITS Dental College, Greater Noida, IND; 5 Department of Prosthodontics, Guru Gobind Singh College of Dental Sciences and Research Centre, Burhanpur, IND; 6 Department of Orthodontics, Kothiwal Dental College and Research Centre, Moradabad, IND

**Keywords:** cone beam computed tomography, crestal, esthetics, gingival former, soft tissue, thickness

## Abstract

Introduction

Accurate selection of gingival formers is critical for achieving optimal peri-implant soft tissue health and esthetics. Crestal mucosal thickness may play a significant role in the success of gingival former selection, particularly in single-tooth implant prosthodontics. The present study aimed to evaluate the impact of crestal mucosal thickness on the accuracy of gingival former selection using retrospective clinical data and cone-beam computed tomography (CBCT) analysis.

Materials and methods

This retrospective, cross-sectional, observational study was conducted in the Department of Prosthodontics after obtaining ethical approval. Patient records of 150 implant sites from August 2021 to December 2024 were reviewed. The inclusion criteria were adults (18-65 years) with single-tooth implants, adequate periodontal health, and complete clinical documentation, including CBCT scans. Patients with systemic conditions, tobacco use, or incomplete medical records were excluded. CBCT images were used to measure crestal mucosal thickness. The gingival former type, diameter, and clinical outcomes were documented. Accuracy of gingival former selection was defined as the absence of inflammation, ulceration, or recession, along with a good emergence profile at the final prosthetic placement. Statistical analyses included chi-square tests, independent t-tests, and multivariate logistic regression.

Results

Of the 150 implant sites, 108 (72%) had accurate gingival former selection, while 42 (28%) showed complications. No significant associations were found for sex (p = 0.894), jaw (p = 0.860), or site (p = 0.138). However, mucosal thickness significantly influenced selection accuracy (p = 0.001), with inaccurate outcomes more common in sites with <2 mm thickness. Accurate selections had significantly thicker mucosa (mean = 2.43 ± 0.3 mm) and smaller gingival formers (mean diameter = 4.45 ± 0.74 mm). Logistic regression revealed that thinner mucosa (odds ratio = 13.92) and larger former diameter (odds ratio = 4.15) were independent predictors of inaccurate selection.

Conclusion

Crestal mucosal thickness is a critical determinant for the accurate selection of gingival formers. Thin soft tissue biotypes and inaccurate formers significantly increase the risk of peri-implant soft tissue complications. Preoperative CBCT assessment and a tissue-informed approach are essential for optimizing outcomes in implant prosthodontics.

## Introduction

The advent of dental implant therapy has significantly enhanced restorative dentistry, offering a reliable and enduring option for tooth replacement [1]. However, the long-term efficacy of implants depends not only on osseointegration but also on the maintenance of peri-implant soft tissue health and esthetic considerations [2]. Judicious selection of the gingival former (healing abutment) is a critical factor that indirectly affects soft tissue healing and contour, as it dictates the configuration of the peri-implant mucosa during the healing phase, subsequently informing the emergence profile of the definitive prosthesis [3].

Therefore, the role of the gingival former should not be underestimated, and selecting an appropriate material is crucial to provide a well-formed and biologically stable mucosal seal around the implant [4]. An appropriate gingival former has been shown to support favorable peri-implant soft tissue outcomes with regard to emergence profile, soft tissue contour, impression-taking, esthetics, and a reduced risk of peri-implant disease [5]. Thus, precise assessment of tissue thickness prior to gingival former selection is essential for optimal functional and esthetic implant rehabilitation.

The final esthetic outcome and biological integrity of the peri-implant mucosa are influenced by soft tissue thickness, particularly in the buccolingual and vertical dimensions [4]. Increased soft tissue thickness has been associated with enhanced esthetic results and reduced mucosal recession. Knowledge of this parameter can assist clinicians in selecting the appropriate height and contour of the gingival former, thereby providing adequate support to the soft tissue during the healing phase. Various methodologies have been employed to estimate soft tissue thickness, including direct probing during surgical procedures, radiographic techniques, and cone-beam computed tomography (CBCT) alongside soft tissue simulation, although the practical application of these methods in daily clinical practice may vary significantly [6].

Despite the recognized importance of soft tissue thickness and gingival formers, limited retrospective studies have specifically examined the correlation between estimated soft tissue thickness, the selection of gingival formers, and the subsequent impact on peri-implant soft tissue outcomes [7,8]. Understanding this relationship is particularly valuable for developing evidence-based protocols for implant site preparation and healing abutment selection, especially in cases where patient-specific anatomical variations influence the soft tissue response. This retrospective study aimed to evaluate the significance of estimating soft tissue thickness in the selection of gingival formers during dental implant placement and to assess the clinical outcomes associated with correct versus incorrect selection of gingival formers. By analyzing clinical records, implant success parameters, and soft tissue responses over a defined follow-up period, this study sought to establish a clearer understanding of how proper selection based on soft tissue estimation can improve implant esthetics, prosthetic outcomes, and long-term peri-implant health.

## Materials and methods

Study design

This retrospective, cross-sectional, observational study was conducted in the Department of Prosthodontics, Jawahar Medical Foundation's Annasaheb Chudaman Patil Memorial Dental College, Dhule, following approval from the Institutional Ethics Committee (ST2024-130). The study involved a review of records of patients who underwent dental implant placement between August 2021 and June 2024. Written informed consent was obtained from all patients as a standard procedure within our department for the use of their records for research purposes, while ensuring the preservation of confidentiality. This study adhered to the principles of the Declaration of Helsinki.

Sample size calculation

The sample size was calculated using G*Power 3.1.9.7.3 (Heinrich-Heine-Universität Düsseldorf, Germany) for gingival recession at 3-month intervals at the implant site. Using a calculated effect size of 0.20 from a previous study with a mean difference (MD) of 0.10 (0.27-0.17) and a pooled SD of 0.51, with 80% power and a 5% alpha error (two-tailed), a minimum of 150 implant sites was required [9].

Eligibility criteria

Individuals aged between 18 and 65 years who required the placement of single-tooth dental implants in the anterior or posterior regions of the maxilla or mandible, had a healthy periodontal condition (as evidenced by probing depths ≤3 mm, absence of clinical attachment loss, and no indicators of periodontitis), adequate bone volume for implantation (verified through preoperative CBCT), high-quality CBCT scans at both the pre-operative stage and at the time of gingival former placement, and comprehensive clinical documentation, including specifications regarding the type and dimensions of the gingival formers used, along with a post-placement monitoring period extending to one month prior to the definitive prosthetic phase, were included in the study. Patients were excluded if they had systemic conditions affecting soft tissue healing (such as uncontrolled diabetes or immunosuppression), current smoking or use of smokeless tobacco, incomplete clinical records, metallic restorations or implants adjacent to the study site causing CBCT artifacts, a history of periodontal surgery at the implant site, or if they had undergone prosthetic revisions due to implant failure or peri-implantitis.

Methodology

Data for this study were retrospectively collected from patient records, intraoral and radiographic images, and clinical photographs stored in an institutional database. A total of 150 implant sites (in 150 patients) were included based on the established inclusion and exclusion criteria, after screening records of 432 patients. The recorded variables included demographic data such as age, sex, and systemic health status, along with implant-related information including brand, size (diameter and length), and site of placement (categorized as maxilla or mandible, and further classified into anterior or posterior regions).

**Figure 1:**
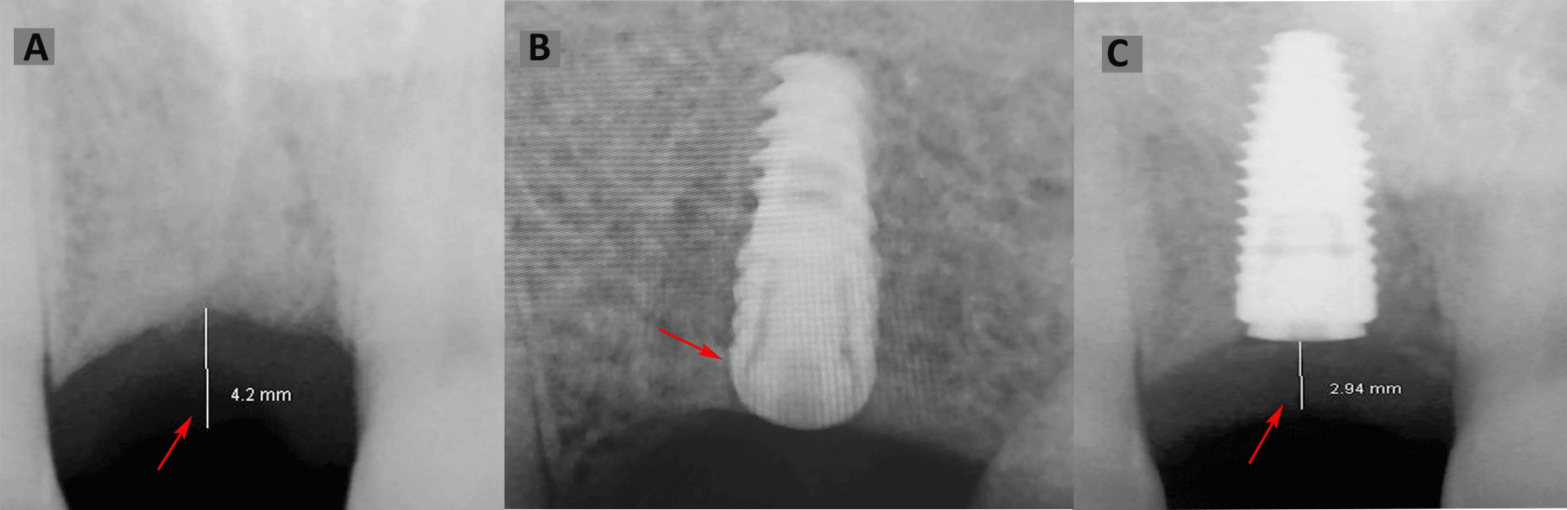
Estimation of crestal mucosal thickness. (A) Preoperative crestal mucosal thickness (in mm) as measured on CBCT; (B) Single-unit implant placement as verified by IOPA; and (C) Postoperative crestal mucosal thickness (in mm) at the third month, measured on CBCT at the time of gingival former placement. CBCT: Cone-beam computed tomography; IOPA: Intraoral periapical radiograph. This figure depicts radiographic images of a patient included in the study.

CBCT scans were utilized for non-invasive soft tissue thickness evaluation, acquired at 80 kVp, 4.3 mA, 17 s, 0.2 mm³ voxel resolution, 0.2 mm slice thickness, and a field of view (FOV) of 4 × 4 cm, using a high-resolution CBCT unit (Planmeca ProMax 3D, Helsinki, Finland). A standardized slice selection protocol using multiplanar reconstruction aligned with the implant’s longitudinal axis was employed, and a metal artifact reduction algorithm was applied to manage implant-related artifacts. CBCT images were analyzed using imaging software to measure the vertical distance from the alveolar bone crest to the outer mucosal surface, ensuring accuracy by selecting cross-sectional slices perpendicular to the alveolar ridge in the mid-buccal region of the implant site (Figure 1).

All included cases involved single-unit dental implant placements (NobelActive, On1™, Zurich, Switzerland) in either the maxillary or mandibular arch, following a conventional two-stage surgical protocol. The implant sites were evaluated clinically and radiographically prior to surgery for adequate bone volume and quality using CBCT. Under local anesthesia and aseptic conditions, implants were placed in healed ridges using a standard surgical protocol recommended by the respective implant system. The position of the implant was verified using intraoral periapical radiographs (IOPA). After achieving primary stability, cover screws were placed and the surgical sites were closed with resorbable sutures. Patients were recalled after a healing period of 3-4 months, depending on the implant site and bone quality. During the second-stage surgery, a mid-crestal incision was made to expose the implant, and the cover screw was replaced with a gingival former (healing abutment). The type and dimensions (height and diameter) of the gingival former were recorded. The soft tissue was allowed to heal and adapt to the gingival former for 3 to 4 weeks. After this healing phase, patients were recalled for the prosthetic phase, during which implant-level impressions were made, followed by the fabrication and placement of the definitive prosthesis.

Soft tissue crestal mucosal thickness was evaluated by CBCT during the preoperative period and at the time of gingival former placement (postoperative period). Clinical outcomes evaluated during the prosthetic phase, which occurred one month after the insertion of the gingival former, included the emergence profile, the occurrence of soft tissue recession or inflammation, and the necessity for recontouring or modification of the gingival former prior to the final impression. Any complications, including soft tissue irritation, ulceration, or delayed healing, were systematically documented. The gingival former was considered accurate if there was no soft tissue inflammation, irritation, or ulceration, and a good emergence profile was observed at the time of final prosthetic placement.

To ensure reliability and standardization in the study, both radiologists (AJ, SM) analyzing the CBCT records underwent calibrated training involving the evaluation of 10 pilot CBCT scans, with an inter-examiner intraclass correlation coefficient (ICC) of ≥ 0.8 considered acceptable for calibration. Blinding was implemented to minimize bias; radiologists interpreting CBCT images were provided with coded data and were unaware of each other’s scores. To further strengthen the blinding process and reduce potential bias, the two evaluators were from different institutions. To enhance reproducibility, CBCT measurements were repeated after a two-week interval for a subset of patients to assess intra-examiner reliability. CBCT scans exhibiting significant motion or metal artifacts were excluded, and imaging parameters were optimized, including appropriate kVp settings, to minimize beam hardening and related artifacts.

Data analysis

Statistical analysis was performed using SPSS (IBM Corp. Released 2013. IBM SPSS Statistics for Windows, Version 23.0. Armonk, NY: IBM Corp.). Shapiro-Wilk tests were used to assess the normality of continuous variables (age, post-implant mucosal thickness, and gingival former diameter). Chi-square tests were used to compare categorical predictors (sex, jaw, and site) between the accurate and inaccurate gingival former selection groups. Independent t-tests were used to evaluate continuous predictors. A multivariate logistic regression model was used to identify predictors of inaccurate selection, including variables significant in univariate analyses, to estimate adjusted OR and 95% CI. Significance was set at p < 0.05. To further minimize analytical bias, statistical analysis was performed by an independent statistician (NP) affiliated with a different institution, who was blinded to the clinical outcomes.

## Results

The sample consisted of 87 (58%) males and 63 (42%) females, with a mean age of 38 years. Analysis of the 150 implant sites in 150 patients revealed no significant association between gingival former selection accuracy and sex (p = 0.894), jaw (p = 0.860), or site (p = 0.138). However, crestal mucosal thickness significantly influenced outcomes (p = 0.001), with inaccurate selections occurring more frequently at sites with <2 mm thickness than at those with >2 mm. All 108 (72%) sites with good responses were classified as accurate, whereas all 42 (28%) sites with thinning or recession were classified as inaccurate (p = 0.001). These findings suggest that crestal mucosal thickness is a critical determinant of gingival former selection accuracy, with thinner mucosa (<2 mm) predisposing patients to complications such as recession and thinning. Sex, jaw, and site appear to be less influential, indicating that tissue thickness should guide former selection to optimize peri-implant mucosal health and minimize adverse outcomes in implant prosthodontics (Table 1).

**Table 1:** Univariate analysis of single implant sites using the chi-square test of association. *p < 0.05: statistically significant. Data are presented as N (%).

Variables	Category	Total N (%)	Accurate N (%)	Inaccurate N (%)	Chi-square	P-value
Sex	Male	87 (58%)	63 (42%)	24 (16%)	0.02	0.894
	Female	63 (42%)	45 (30%)	18 (12%)		
Jaw	Upper	66 (44%)	48 (32%)	18 (12%)	0.03	0.86
	Lower	84 (56%)	60 (40%)	24 (16%)		
Site	Anterior	57 (38%)	45 (30%)	12 (8%)	2.2	0.138
	Posterior	93 (62%)	63 (42%)	30 (20%)		
Response	Good	108 (72%)	108 (72%)	0 (0%)	150	0.001*
	Thinning	24 (16%)	0 (0%)	24 (16%)		
	Recession	18 (12%)	0 (0%)	18 (12%)		
Crestal mucosa thickness (mm)	< 2 mm	36 (24%)	11 (7%)	25 (17%)	42.29	0.001*
	≥ 2 mm	114 (76%)	97 (65%)	17 (11%)		

Independent t-tests were used to compare crestal mucosal thickness, gingival former diameter, and age between accurate and inaccurate gingival former selections. Accurate selections exhibited significantly thicker crestal mucosa (mean = 2.43 ± 0.3 mm) compared to inaccurate sites (mean = 1.91 ± 0.29 mm). The gingival former diameter was significantly smaller in accurate cases (mean = 4.45 ± 0.74 mm) than in inaccurate ones (mean = 5.04 ± 0.54 mm). There were no significant differences in age between the groups (p = 0.986). These findings suggest that thinner crestal mucosa and larger gingival former diameters are associated with inaccurate selection and an increased risk of recession and thinning. Accurate selection requires matching smaller diameters to thicker mucosa, whereas age appears unrelated to outcomes (Table 2).

**Table 2:** Comparison of outcomes (accurate vs. inaccurate gingival former size) for independent variables using independent t-test. *p < 0.05: statistically significant; Data are presented as mean ± SD.

Variables	Outcome	95% CI for Mean	Mean ± SD	t-value	p-value
Crestal mucosal thickness (mm)	Accurate	2.37-2.48	2.43 ± 0.30	9.62	0.001*
	Inaccurate	1.82-2.00	1.91 ± 0.29		
Gingival former diameter (mm)	Accurate	4.31-4.59	4.45 ± 0.74	4.65	0.001*
	Inaccurate	4.87-5.21	5.04 ± 0.54		
Age (years)	Accurate	37.24-40.38	38.81 ± 8.25	0.02	0.986
	Inaccurate	36.20-41.36	38.78 ± 8.17		

Crestal mucosal thickness was inversely associated with inaccurate selection (OR = 1.43), indicating that thicker mucosa reduced the odds of inaccuracy. Conversely, a larger gingival former diameter significantly increased the likelihood of inaccurate selection (OR = 4.15), with each 1 mm increase elevating the odds fourfold. Sites with crestal mucosa thickness <2 mm had markedly higher odds of inaccurate selection (OR = 13.92). These findings suggest that thinner mucosa, particularly <2 mm, and larger former diameters are strong independent predictors of inaccurate gingival former selection, contributing to complications such as recession and thinning. Clinicians should prioritize smaller formers for thin mucosa to optimize peri-implant outcomes, aligning with the higher complication rates observed in such cases (Table 3).

**Table 3:** Multivariate logistic regression analysis for the occurrence of inaccurate gingival former selection. *p < 0.05: statistically significant.

Variables	Coefficient B	Standard error	z-value	P-value	Odds Ratio	95% CI
Crestal mucosal thickness (mm)	-5.38	0.89	6.05	0.001*	1.43	0.96-2.03
Gingival former diameter (mm)	1.42	0.35	4.07	0.001*	4.15	2.09-8.23
Crestal mucosal thickness (< 2 mm)	2.63	0.45	5.84	0.001*	13.92	5.75-33.71

## Discussion

The present study assessed the association between crestal mucosal thickness and the accuracy of gingival former selection in patients undergoing single-tooth implant placement. The findings underscore the significance of the peri-implant soft tissue biotype, particularly crestal mucosal thickness, in determining the success and stability of soft tissue outcomes following implant placement.

One of the primary observations in this study was the statistically significant association between thinner crestal mucosa (<2 mm) and an increased risk of inaccurate gingival former selection. Empirical investigations have demonstrated that the breadth of keratinized mucosa plays a critical role in maintaining peri-implant health, revealing significant correlations between crestal bone resorption and insufficient keratinized tissue, particularly in the presence of inflammatory conditions [10,11]. A limited width of keratinized mucosa is associated with plaque accumulation, onset of inflammation, and mucosal tissue recession, which may consequently exacerbate crestal bone loss [12]. Systematic reviews have shown that implants surrounded by minimal soft tissue experience higher incidences of crestal bone resorption within the first 12 months of functional loading [13,14]. These findings indicated that soft tissue thickness did not influence crestal bone loss after the initial 12 months of loading. In a systematic review encompassing six clinical investigations, four studies reported significantly greater initial crestal bone loss at implant sites with soft tissue thickness <2 mm, whereas no statistically significant differences were observed in the remaining two studies [13]. The delicate and less resilient nature of thin mucosa may not accommodate larger gingival formers effectively, leading to soft tissue irritation, inflammation, and eventual recession.

The significance of gingival former diameter in relation to tissue thickness also emerged as a critical factor in this study. Larger gingival former diameters were strongly associated with inaccurate selection and complications, particularly in cases with thin mucosa. An OR of 4.15 per mm increase in diameter highlights the potential for disproportionately large formers to exert excessive pressure on the peri-implant mucosa, thereby compromising vascular supply and inducing soft tissue breakdown. These findings support the clinical notion that gingival formers should be selected not only based on the final prosthetic design but also in accordance with the existing soft tissue architecture.

Interestingly, variables such as sex, age, jaw (maxilla versus mandible), and site location (anterior versus posterior) showed no statistically significant influence on the accuracy of gingival former selection. This contrasts with earlier studies that reported variations in soft tissue response between maxillary and mandibular implants or between anterior and posterior sites [15,16]. The lack of significance in the present study could be attributed to standardized surgical protocols, the exclusion of systemic factors and periodontal disease, and the controlled environment in which the implant surgeries were performed. Moreover, consistency in clinician training and prosthetic workflows may have contributed to minimizing the variability typically introduced by anatomical site differences.

We used 2.0 mm as a cut-off point to differentiate between thin and thick tissue, in accordance with previous clinical studies [17,18]. Logistic regression analysis provided deeper insights into the independent determinants influencing the accuracy of gingival former selection. Sites exhibiting crestal mucosal thickness <2 mm demonstrated an almost 14-fold increase in the probability of erroneous selection, thereby underscoring the critical importance of mucosal thickness in clinical decision-making. The inverse correlation observed between mucosal thickness and inaccurate gingival former selection further reinforces the premise that thinner biotypes exhibit diminished tolerance to soft tissue manipulation and prosthetic interventions. This finding has direct implications for prosthodontic planning, where a "one-size-fits-all" approach to former selection is inadequate. Instead, the implementation of a patient-specific strategy, incorporating CBCT-based mucosal evaluations, should be adopted to optimize clinical outcomes.

CBCT imaging played a crucial role in this study by enabling accurate and non-invasive measurement of soft tissue thickness. The use of high-resolution parameters, combined with stringent calibration and reliability protocols, enhanced the validity of the measurements. Previous studies have validated CBCT as a reliable tool for evaluating both hard and soft tissues in implant dentistry [6,19]. However, certain limitations, such as beam-hardening artifacts, especially in the presence of metallic restorations, can affect image interpretation. In this study, these limitations were mitigated through artifact exclusion criteria and optimized imaging parameters. The methodological rigor of CBCT analysis ensured reproducibility and reliability of the mucosal measurements, which served as a cornerstone of the study’s outcomes.

Clinical implications

The clinical implications of these findings are multi-faceted. First, preoperative evaluation of soft tissue biotypes using CBCT should be established as a standard procedure in dental implant planning. This evaluation facilitates anticipation of possible complications and allows customization of gingival formers to align with the unique anatomical characteristics of individual patients. Second, gingival former selection should be adaptable, taking into account the dynamic tissue response during the healing process. In cases of a thin biotype, initiating treatment with a narrower and shorter gingival former, and progressively advancing toward the desired contour, may reduce tissue trauma and support favorable development of the emergence profile. Moreover, this study emphasizes the need for interdisciplinary coordination between surgeons and prosthodontists. Effective communication regarding tissue characteristics, implant positioning, and prosthetic requirements can facilitate the selection of appropriate healing abutments that support soft tissue maturation while minimizing adverse events. Incorporating digital workflows and 3D-printed customized healing abutments may further enhance the precision and patient-specific adaptation of soft tissue contours.

Limitations

Although this study possesses certain strengths, such as a meticulously calibrated and standardized methodology alongside comprehensive statistical analysis, it also has notable limitations. The retrospective design inherently restricts control over confounding variables and limits the ability to establish causal relationships. Despite the implementation of stringent inclusion and exclusion criteria, potential biases such as variations in operator experience, subtle differences in surgical technique, and individualized patient healing responses may have influenced the outcomes observed. The follow-up duration was limited to the period preceding the placement of the definitive prosthesis, thereby precluding the evaluation of long-term soft tissue stability post-restoration. Future research employing prospective designs, longer follow-up durations, and histological validation of mucosal thickness may provide more comprehensive insights into this area.

## Conclusions

This study highlights the pivotal role of crestal mucosal thickness in determining the accuracy of gingival former selection in single-tooth implant prosthodontics. Thinner soft tissue biotypes (<2 mm) were significantly associated with inaccurate gingival former selection, leading to higher incidences of soft tissue complications such as recession and mucosal thinning. In contrast, sites with thicker mucosa demonstrated improved soft tissue responses and more favorable emergence profiles. Additionally, larger gingival former diameters were independently associated with increased odds of selection inaccuracy, particularly in sites with thin tissue. Other factors, such as sex, age, jaw, and implant site location, did not significantly influence the outcomes. Based on these findings, clinicians are encouraged to adopt a tissue-driven approach, selecting smaller formers for thin biotypes to minimize complications and enhance peri-implant soft tissue stability and esthetics.

## References

[1] Shayya G, Benedetti C, Chagot L, Stachowicz ML, Chassande O, Catros S (2024). Revolutionizing dental implant research: a systematic review on three-dimensional in vitro models. Tissue Eng Part C Methods.

[2] Sharma M, Bhatia T, Sharma R, Nandan R, Bagga A, Kapoor H (2024). Bridging the gap: soft tissue considerations in dental implants from microscopic to macroscopic levels. Cureus.

[3] Son MK, Jang HS (2011). Gingival recontouring by provisional implant restoration for optimal emergence profile: report of two cases. J Periodontal Implant Sci.

[4] Jose EP, Paul P, Reche A (2023). Soft tissue management around the dental implant: a comprehensive review. Cureus.

[5] Wang Y, Zhang Y, Miron RJ (2016). Health, maintenance, and recovery of soft tissues around implants. Clin Implant Dent Relat Res.

[6] Gürlek Ö, Sönmez Ş, Güneri P, Nizam N (2018). A novel soft tissue thickness measuring method using cone beam computed tomography. J Esthet Restor Dent.

[7] Bienz SP, Pirc M, Papageorgiou SN, Jung RE, Thoma DS (2022). The influence of thin as compared to thick peri-implant soft tissues on aesthetic outcomes: a systematic review and meta-analysis. Clin Oral Implants Res.

[8] Groenendijk E, Bronkhorst EM, Meijer GJ (2021). Does the pre-operative buccal soft tissue level at teeth or gingival phenotype dictate the aesthetic outcome after flapless immediate implant placement and provisionalization? Analysis of a prospective clinical case series. Int J Implant Dent.

[9] Ross SB, Pette GA, Parker WB, Hardigan P (2014). Gingival margin changes in maxillary anterior sites after single immediate implant placement and provisionalization: a 5-year retrospective study of 47 patients. Int J Oral Maxillofac Implants.

[10] Lin GH, Chan HL, Wang HL (2013). The significance of keratinized mucosa on implant health: a systematic review. J Periodontol.

[11] Thöne-Mühling M, Kelm D, Mengel R (2016). Width of keratinized mucosa at implant sites in patients treated for generalized aggressive periodontitis: a cohort study. Int J Oral Maxillofac Implants.

[12] Breunig N, Stiller M, Mogk M, Mengel R (2024). Influence of gingival phenotype on crestal bone loss at implants: a long-term 2 to 20-year cohort study in periodontally compromised patient. Int J Implant Dent.

[13] Akcalı A, Trullenque-Eriksson A, Sun C, Petrie A, Nibali L, Donos N (2017). What is the effect of soft tissue thickness on crestal bone loss around dental implants? A systematic review. Clin Oral Implants Res.

[14] Suárez-López Del Amo F, Lin GH, Monje A, Galindo-Moreno P, Wang HL (2016). Influence of soft tissue thickness on peri-implant marginal bone loss: a systematic review and meta-analysis. J Periodontol.

[15] Cui X, Reason T, Pardi V, Wu Q, Martinez Luna AA (2022). CBCT analysis of crestal soft tissue thickness before implant placement and its relationship with cortical bone thickness. BMC Oral Health.

[16] Braut V, Bornstein MM, Belser U, Buser D (2011). Thickness of the anterior maxillary facial bone wall-a retrospective radiographic study using cone beam computed tomography. Int J Periodontics Restorative Dent.

[17] Linkevicius T, Apse P, Grybauskas S, Puisys A (2010). Influence of thin mucosal tissues on crestal bone stability around implants with platform switching: a 1-year pilot study. J Oral Maxillofac Surg.

[18] Linkevicius T, Apse P, Grybauskas S, Puisys A (2009). The influence of soft tissue thickness on crestal bone changes around implants: a 1-year prospective controlled clinical trial. Int J Oral Maxillofac Implants.

[19] Ferry K, AlQallaf H, Blanchard S, Dutra V, Lin WS, Hamada Y (2022). Evaluation of the accuracy of soft tissue thickness measurements with three different methodologies: an in vitro study. J Periodontol.

